# *Loxosceles niedeguidonae* (Araneae, Sicariidae) a new species of brown spider from Brazilian semi-arid region

**DOI:** 10.3897/zookeys.175.2259

**Published:** 2012-03-16

**Authors:** Rute Maria Gonçalves-de-Andrade, Rogério Bertani, Roberto Hiroaki Nagahama, Maria Fatima Ribeiro Barbosa

**Affiliations:** 1 Laboratório de Imunoquímica; 2 Laboratório Especial de Ecologia e Evolução, Instituto Butantan; Av. Vital Brazil 1500, São Paulo, São Paulo, 05503-900, Brazil; 3 Fundação Museu do Homem Americano, São Raimundo Nonato, Piauí, Brazil; 4 Universidade Federal do Vale do São Francisco, São Raimundo Nonato, Piauí, Brazil

**Keywords:** Brown spider, Semi-arid region, Piauí, Taxonomy, Araneae, Sicariidae, *Loxosceles*, *Loxosceles niedeguidonae*

## Abstract

A new species of recluse spider, *Loxosceles niedeguidonae* **sp. n.**, is described from the Parque Nacional Serra da Capivara, State of Piauí, Brazil. This is the first endemic species described from Brazilian semi-arid environment. The species is included in *gaucho* group of Gertsch (1967) due to its spermathecal shape and is considered close to *Loxosceles chapadensis* Bertani, Fukushima & Nagahama, 2010 by the unusual long male palpal tibia, a character not common for species of this group. An updated key for *Loxosceles* species of *gaucho* group is presented.

## Introduction

The *Loxosceles* Heineken & Lowe, 1832 species, brown recluse spiders, are spread throughout the world. Currently, there are 102 species described for the whole Neotropical region, Europe, Asia and Africa, eleven of them present and/or endemic to Brazil (Gertsch 1967; Gertsch and Ennik 1983; Bertani et al. 2010; Platnick 2011).

In general, for the classified species of this genus the authors have adopted the species groups proposed by Gertsch (1967), based mainly on characteristics of the female genitalia and the male copulatory organ. This author proposed four species groups for South America: *amazonica* group – with a single species, *Loxosceles amazonica* Gertsch, 1967 (Brazil) – characterized by males having a palpal tarsus considerably shorter than the tibia, and the females having spermathecae with a group of small, globular lobes at the apex; *gaucho* group - with five species, *Loxosceles adelaida* Gertsch, 1967 (Brazil), *Loxosceles gaucho* Gertsch, 1967 (Brazil, Tunisia), *Loxosceles similis* Moenkhaus, 1898 (Brazil), *Loxosceles variegata* Simon, 1897 (Paraguay) and recently described Brazilian species *Loxosceles chapadensis* Bertani, Fukushima & Nagahama, 2010 - the tibia and palpal tarsus of males is equivalent in length (except *Loxosceles chapadensis*) and females have a crosswise sclerotized plate connected to the spermathecae; *laeta* group - with 24 species – American widespread species *Loxosceles laeta* (Nicolet, 1849) (New World, introduced in Finland and Australia); fourteenPeruvian species *Loxosceles accepta* Gertsch, 1967, *Loxosceles alicea* Gertsch, 1967, *Loxosceles bettyae* Gertsch, 1967, *Loxosceles blancasi* Gertsch, 1967, *Loxosceles conococha* Gertsch, 1967, *Loxosceles frizzelli* Gertsch, 1967, *Loxosceles harrietae* Gertsch, 1967, *Loxosceles herreri* Gertsch, 1967, *Loxosceles inca* Gertsch, 1967, *Loxosceles julia* Gertsch, 1967, *Loxosceles olmea* Gertsch, 1967, *Loxosceles piura* Gertsch, 1967, *Loxosceles pucara* Gertsch, 1967, *Loxosceles surca* Gertsch, 1967; *Loxosceles gloria* Gertsch, 1967 (Peru e Ecuador), *Loxosceles taeniopalpis* Simon, 1907 (Ecuador), *Loxosceles lutea* Keyserling, 1877 (Colombia, Ecuador), *Loxosceles rufipes* (Lucas, 1834) (Guatemala, Panama, Colombia), *Loxosceles lawrencei* Caporiacco, 1955 (Venezuela, Trinidad, Curaçao), *Loxosceles panama* Gertsch, 1958 (Panama), *Loxosceles coquimbo* Gertsch, 1967 (Chile) and Brazilian species *Loxosceles puortoi* Martins, Knysak & Bertani, 2002 - male palpal tibia at least two times longer than tarsus, whereas in females the spermathecae vary, but in general they are long with receptacles nearby and free; *spadicea* group - *Loxosceles hirsuta*Mello-Leitão, 1931 (Brazil, Paraguay, Argentina), *Loxosceles intermedia* Mello-Leitão, 1934 (Brazil, Argentina)and *Loxosceles spadicea* Simon, 1907 (Peru, Bolivia, Argentina) – males have a spherical palpal bulb and a thin embolus with a carina at its base and females have well-separated spermathecae with small epigynum ducts.

After Gertsch (1967), and despite the medical importance of the genus, little more has been added to the taxonomy of the South-American *Loxosceles*. Brignoli (1978) transferred *Calheirosa anomala* Mello-Leitão, 1917 and *Calheirosa immodesta* Mello-Leitão, 1917 to the genus *Loxosceles*. Álvares et al. (2004) redescribed *Loxosceles anomala* (Mello-Leitão, 1917) and considered this species to belong to the *spadicea* group. Other authors have expanded the knowledge about the geographic distribution of some species (Gonçalves-de-Andrade et al. 2001, 2007; Gonçalves-de-Andrade and Tambourgi 2003; Silveira 2009). In the last decade two new species were described: *Loxosceles puortoi* considered to belong to *laeta* group, the first species of this group endemic to Brazil and, more recently (2010), *Loxosceles chapadensis*, which was included in the *gaucho* group.

The Parque Nacional Serra da Capivara is a federal protected area in Southeastern State of Piauí, Brazil. The 129,953 ha cover areas in the municipalities of São Raimundo Nonato, Coronel José Dias, João Costa and Brejo do Piauí (08°26', 08°54'S and 42°19', 42°45'W). Together with Parque Nacional Serra das Confusões it is one of the largest protected areas in the world with “Caatinga” vegetation formation, situated between the “Médio São Francisco” depression (Precambriam) and the Piauí–Maranhão sedimentary basin (Silurian-Devonian), the two largest geological formations in Brazilian Northeastern. Rainfall period is from November to March, with a yearly median precipitation of 689 mm. The annual median temperature is 28°C. General landscape of Parque Nacional da Serra da Capivara consists of uplands, plateaus, hills, mountain chains and plains. This distinct relief is a result of transformations occurring during millions of years in the sedimentar basin of Piauí–Maranhão and in the central São Francisco Depression composed of different types of minerals and rocks.

In this work we describe a new species of *Loxosceles* endemic to the Brazilian semiarid environment, from Parque Nacional Serra da Capivara, the second species of the *gaucho* group described for Northeastern Brazil and present an updated key for *Loxosceles* species of *gaucho* group.

### Material and methods

Spiders were collected (the permissions for collect - number 11971-2 - and for work in Parque Nacional Serra da Capivara – number 18413-1 - was conceded to Rute Maria Gonçalves-de-Andrade by ICMBio a instance of the Brazilian Ministry of the Environment) in two localities: Toca do Buraco da Pedra Furada and Boqueirão do Gato. The specimens examined are deposited in Museu Nacional, Rio de Janeiro, RJ, Brazil (Dr. Adriano B. Kury). The copulatory organs of females were dissected and cleared with clove oil. A LEICA® MZ7.5 Stereomicroscope with 10x eyepiece was used for illustrations (with a camera lucida attachment) and measurements (using an ocular micrometer). Measurements are in millimeters. Abbreviations: ALE = anterior lateral eye, PLE = posterior lateral eye and PME = posterior median eye.

## Taxonomy

### Loxosceles

Heineken & Lowe, 1832

http://species-id.net/wiki/Loxosceles

#### Identification key for species of Loxosceles of gaucho group

 [Modified from Gertsch (1967)]

**Table:** 

1	Males	2
–	Females	7
2	Palpal tibia more than 2 times longer than palpal tarsus	3
–	Palpal tibia at most 1.5 times longer than palpal tarsus	4
3	Palpal tarsus almost 2 times longer than wide (Figs 2–4); lateral dentate dark bands on the dorsal side of the carapace faded (Fig. 9)	*Loxosceles niedeguidonae* sp. n.
–	Palpal tarsus less than 1.5 times longer than wide; lateral dentate dark bands on dorsal side of the carapace conspicuous (Fig. 10)	*Loxosceles chapadensis*
4	First femur 2.1 times, first leg more than seven times longer than carapace	5
–	First femur at most 1.7 times, first leg at most seven times longer than carapace	6
5	Embolus straight in ventral view (Brignoli 1972, fig. 1)	*Loxosceles adelaida*
–	Embolus strongly curved in ventral view (Gertsch 1967 pl. 4, fig. 4)	*Loxosceles similis*
6	Femur of palpus 3 times longer than wide (Gertsch 1967 pl. 4, fig. 1)	*Loxosceles gaucho*
–	Femur of palpus 5 times longer than wide (Gertsch 1967 pl. 4, fig. 6)	*Loxosceles variegata*
7	Palpal tarsus incrassated and broader than palpal tibia (Fig. 6)	8
–	Tarsus evenly tapered	9
8	Seminal receptacles inconspicuous	*Loxosceles adelaida*
–	Seminal receptacles conspicuous (Figs 7–8)	*Loxosceles niedeguidonae* sp. n.
9	Seminal receptacles small, oval pouches (Gertsch 1967 pl.3, fig. 9)	*Loxosceles variegata*
–	Seminal receptacles much larger	10
10	Seminal receptacles curved (Gertsch 1967 pl.3, fig. 5); first femur 1.3 times longer than carapace	*Loxosceles gaucho*
–	Seminal receptacle straight or almost so; first femur 1.8 times longer than carapace	11
11	Dorsal part of the bursa copulatrix weakly sclerotized (Gertsch 1967 pl. 3, fig. 6)	*Loxosceles similis*
–	Dorsal part of the bursa copulatrix strongly sclerotized (Bertani et al. 2010, figs 6–7)	*Loxosceles chapadensis*

### Loxosceles niedeguidonae sp. n.

urn:lsid:zoobank.org:act:ED9F0A10-B566-4989-AB71-00E1F96BD38E

http://species-id.net/wiki/Loxosceles_niedeguidonae

**Figures 1–8.**
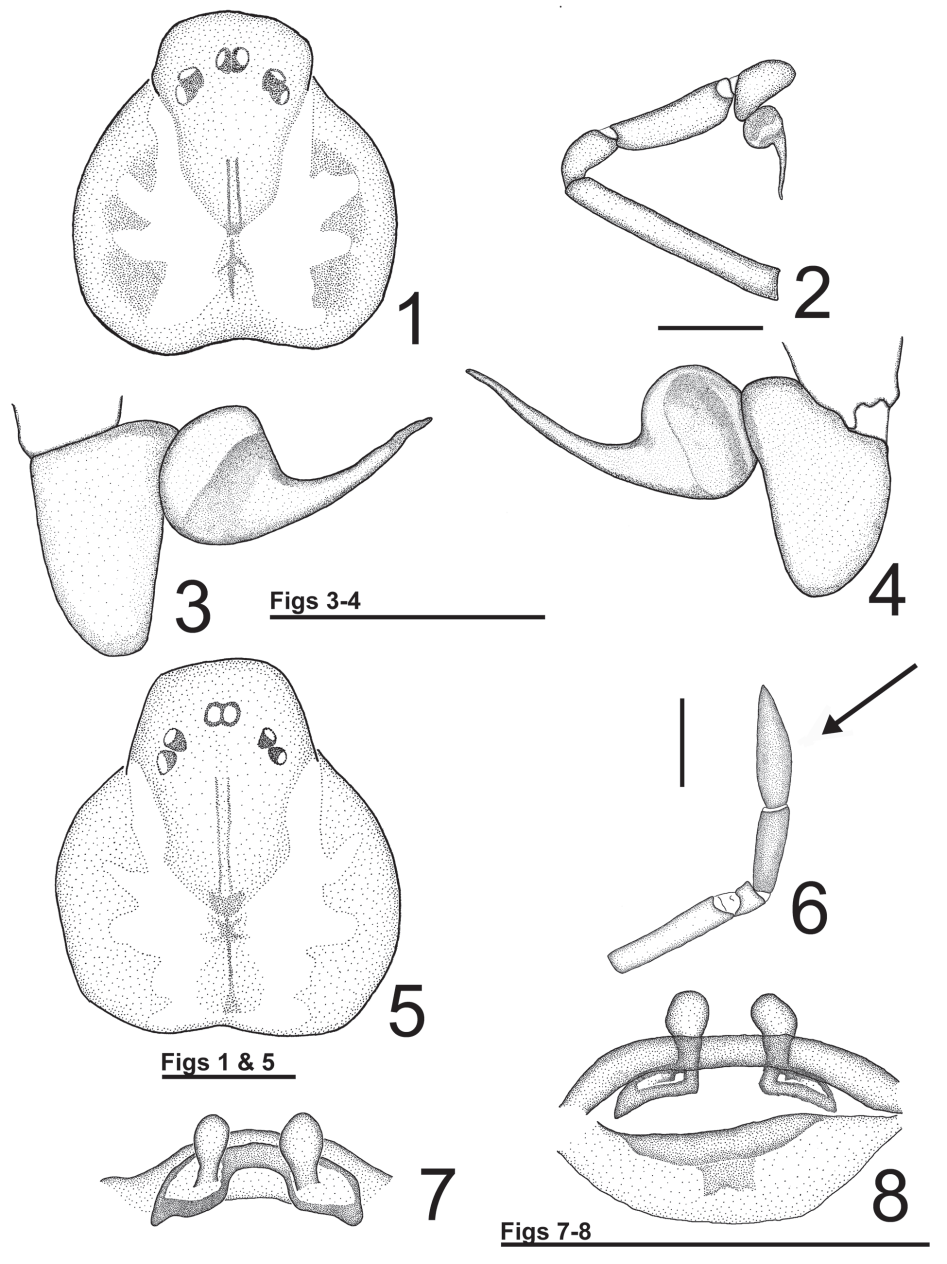
*Loxosceles niedeguidonae* sp. n. **1–4** holotype male **1** carapace **2** left palp **3** bulb, prolateral view **4** bulb, retrolateral view **5–8** paratype female **5** carapace **6** right palp - incrassate tarsus (arrow) **7** spermathecae, dorsal view **8** spermathecae, ventral view. Scale bar: 1 mm.

Figures 1 [Fig F2] –13

#### Type material.

Holotype: male: Brazil, Piauí, Coronel José Dias, Boqueirão do Sítio da Pedra Furada – Toca, 8°51'S, 42°33'W, 16 March 2009, R. M. Gonçalves-de-Andrade & Maria Fátima Ribeiro Barbosa (MNRJ 04359).

Paratype female (MNRJ 04360) with same data as for holotype.

#### Other material examined.

*Loxosceles chapadensis*. Holotype: Male: Brazil, Bahia, Palmeiras, Chapada Diamantina National Park (12°28'S, 41°25'W), 15 February 2008, R. Bertani, C. S. Fukushima & R. H. Nagahama, (MNRJ 6047); Paratypes: Brazil, Bahia*:* 1 female, with same data as for holotype (MNRJ 6048); 3 females and 1 male, Lençóis, Chapada Diamantina National Park (12°33'S, 41°23'W), 19 February 2008, same collectors as for holotype, (MNRJ 6049); 1 female, Iraquara, Fazenda Pra- tinha (12°21'S, 41°32'W), 16.II.2008, same collectors as for holotype, (MNRJ 6050).

#### Diagnosis.

**Figures 9–10. F2:**
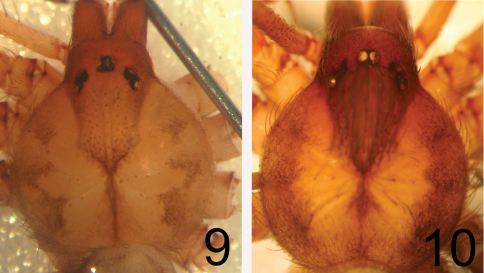
Carapaces, males. **9** *Loxosceles niedeguidonae* sp. n., holotype **10** *Loxosceles chapadensis*, holotype.

Males can be readily distinguished from other species, except *Loxosceles chapadensis* by the following characters in combination: Palpal tibia more than 1.5 times longer than tarsus, embolus 1.5–1.8 times longer than diameter of tegulum, arising at distal half of tegulum, distally thin but not filiform and hardly curved, particularly not in distal direction (Fig. 2). Males differ from *Loxosceles chapadensis* by palpal tarsus almost two times longer than wide (Figs 2–4), embolus less curved (Fig. 4) and faded lateral dentate dark bands on the dorsal side of the carapace (Fig. 9). Females can be recognized by the following characters in combination: A narrow transversal plate in the spermathecae, straight, apically enlarged seminal receptacles and incrassate palpal tarsus, which is broader than palpal tibia (Figs 6–8).

#### Etymology.

The specific name is a patronym in honor of Dr Niéde Guidon, one of the most important Brazilian archeologists, internationally acknowledged for her archeological work and her battle for the preservation of archeological sites in Brazil and the conservation of remnant patches of Caatinga vegetation, as well as important social work, especially in the Parque Nacional da Serra da Capivara.

#### Description.

Male(holotype). Total length (without chelicerae) 6.01. Carapace 3.29 long, 3.1 wide. Eye size: ALE: 0.2, PME: 0.22, PLE: 0.18. Clypeus: 0.32. Interocular distance - PME and PLE: 0.04, PME and ALE: 0.2. Leg formula: II, IV, I, III. Legs and palp length and width in Table 1. Labium 0.75 long, 0.42 wide. Sternum 1.59 long, 1.48 wide. Femur I 2.58 times longer than the carapace. Palpal femur 7.13 times longer than wide, tibia 2.81 times longer than wide (Fig. 2), tarsus almost two times longer than wide (Figs 3–4). Bulb suboval, slightly shorter than tarsus length. Embolus almost straight, approximately two times longer than bulb width, without carina (Fig. 4). Cephalic region of carapace covered by many long setae. Carapace with pars cephalic and chelicerae light brown (Fig. 1). Pars thoracica pale yellow, carapace border mottled, light brown (Figs 1, 9). Legs and palps pale yellow, covered by short grayish setae. Coxae and sternum pale yellow, labium and endites brown. Abdomen covered with grayish setae.

**Table 1.** *Loxosceles niedeguidonae* sp. n. Male holotype. Length/width of right legs and palpal segments.

	**Palp**	**I**	**II**	**III**	**IV**
Tarsi	0.75/0.46	1.9/0.1	1.75/0.12	1.37/0.12	1.75/0.12
Metatarsi	---	11.4/0.2	13.87/0.12	2.05/0.25	12.12/0.25
Tibiae	1.55/0.55	10.1/0.3	12.12/0.37	7.62/0.25	9.37/0.37
Patellae	0.7/0.45	1.3/0.4	1.25/0.5	1.25/0.5	1.25/0.5
Femora	2.64/0.37	8.5/0.6	10.25/0.62	8.0/0.62	9.12/0.62
Total length	5.64	33.2	39.24	20.29	33.61

Female (paratype MNRJ 04360) Total length (without chelicerae) 7.35. Carapace 3.79 long, 3.71 wide. Eye sizes: ALE: 0.22, PME: 0.16, PLE: 0.18. Clypeus: 0.32. Interocular distances - PME and PLE: 0.05, PME and ALE: 0.24. Leg formula: II, I, VI, III. Legs and palp length and width in Table 2. Labium 0.7 long, 0.54 wide. Sternum 1.85 long, 1.78 wide. Femur I 1.58 times longer than carapace. Palpal femur 5.0 times longer than wide, tibia 3.5 longer than wide, tarsus incrassate (Fig. 6). Spermathecae with long, straight, apically enlarged seminal receptacles; transversal plate narrow, weakly sclerotized; atriobursal orifices well visible, ovals, positioned on the internal edge of the central windows; dorsal part of bursa copulatrix weakly sclero- tized (Figs 7–8). Coloration as in male, but darker (Fig. 12). Tarsi and tibiae of palps reddish-brown.

**Table 2.** *Loxosceles niedeguidonae* sp. n. Female paratype (MNRJ 04360). Length/width of right legs and palpal segments.

	**Palp**	**I**	**II**	**III**	**IV**
Tarsi	1.42/0.39	1.58/0.08	1.58/0.16	1.18/0.16	1.26/0.16
Metatarsi	---	6.24/0.16	7.19/0.16	5.85/0.16	6.95/0.16
Tibiae	1.11/0.32	6.32/0.32	6.79/0.32	4.42/0.32	5.45/0.32
Patellae	0.47/0.32	1.11/0.47	1.03/0.32	1.03/0.39	1.11/0.47
Femora	1.58/0.32	6.0/0.63	6.4/0.63	5.45/0.47	5.85/0.63
Total length	4.58	21.25	22.99	17.93	20.62

**Figures 11–14.**
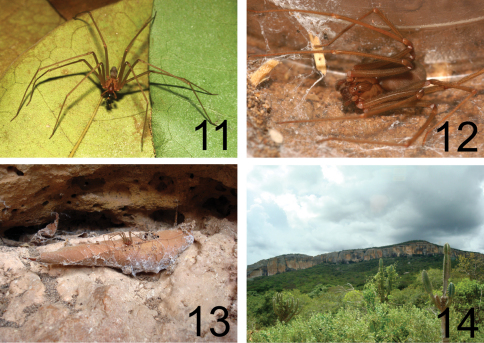
*Loxosceles niedeguidonae* sp. n. **11** male **12** female **13** male in type locality – Toca do Buraco da Pedra Furada, Parque Nacional Serra da Capivara, Piauí, Brazil **14** General view of Parque Nacional Serra da Capivara, Piauí, Brazil. Photos **11–12** R. Bertani; **13–14** R. M. Gonçalves-de-Andrade.

#### Discussion.

The female *Loxosceles niedeguidonae* sp. n. can undoubtedly be included in the *gaucho* group of Gertsch (1967) due to spermathecae bearing a transverse plate (Figs 7–8), which is characteristic of the group. However, the male resembles species of the *laeta* group due to its long palpal tibia (Fig. 2). These characteristics are also found in the recently described species *Loxosceles chapadensis* with which *Loxosceles niedeguidonae* sp. n. seems to be closely related. As already discussed by Bertani et al. 2010, the long palpal tibia of males of *Loxosceles chapadensis* (and now, *Loxosceles niedeguidonae* sp. n.) could be an homoplasy with species of the *laeta* and *spadicea* groups or a plesiomorphy, indicating a more basal position of the species in the *gaucho* group relative to these other species (Bertani et al. 2010). Despite these shared characteristics they can be separated by genitalic and somatic characteristics. The male has a straighter embolus (Figs 3–4), palpal tarsus almost two times longer than wide and color pattern are not very similar to the typical *gaucho* pattern, i. e. lateral dentate dark bands on the dorsal side of carapace. The male resembles an individual of the *laeta* group due to its light-brown coloration and faded dentate dark bands on the carapace (Figs 1–9, 11). The female can be easily separated by the narrow transversal plate in spermathecae (Figs 7–8) and in- crassate palpal tarsus (Fig. 6), which is like the female *Loxosceles adelaida*, *gaucho* group (Gertsch, 1967), but more dilated than the latter.

The distinct, but close geographical distribution of *Loxosceles chapadensis* and *Loxosceles niedeguidonae* sp. n. in Northeastern Brazil provides an additional indication that these two species are closely related.

#### Natural History.

Specimens of *Loxosceles niedeguidonae* sp. n. were found in the locality of “Buraco da Pedra Furada”. The male was collected over the ground and the female in a rock crevice close to a cave mouth. The arenitic walls in the Parque Nacional Serra da Capivara have innumerous crevices that make typical retreats for brown recluse spiders (Figs 13–14). However, despite the presence of so many crevices, a low population density was found in the analyzed ecotope.

#### Distribution.

 Known only from Parque Nacional Serra da Capivara, Piauí State, Brazil.

## Supplementary Material

XML Treatment for Loxosceles

XML Treatment for Loxosceles niedeguidonae

## References

[B1] Álvares ESS Rodrigues T De Maria M (2004) On *Loxosceles anomala* (Mello-Leitão) (Araneae: Sicariidae). Revista Ibérica de Aracnología 10: 293-295.

[B2] Bertani R Fukushima CS Nagahama RH (2010)*Loxosceles chapadensis* (Araneae: Sicariidae): a new recluse spider species of the *gaucho* group from Brazil. The Journal of Arachnology 38: 364–367. http://www.americanarachnology.org/JoA_free/JoA_v38_n2/arac-38-02-364.pdf, doi: 10.1636/A09-92.1

[B3] Brignoli PM (1972) Sur quelques araignées cavernicoles d’Argentine, Uruguay, Brésil et Venezuela récoltées par le Dr P. Strinati. Revue Suisse de Zoologie 79: 361-385.

[B4] Brignoli PM (1978) Spinnen aus Brasilien, II. Vier neue Ochyroceratidae aus Amazonas nebst Bemerkugen über andere Amerikanische Arten (Arachnida: Araneae). Studies on Neotropical Fauna and Environment 13: 11-21. doi: 10.1080/01650527809360529

[B5] Gertsch WJ (1967) The spider genus *Loxosceles* in South America (Araneae, Scytodidae). Bulletin of the American Museum of Natural History 136:117–174. http://digitallibrary.amnh.org/dspace/handle/2246/1989

[B6] Gertsch WJ Ennik F (1983) The spider genus *Loxosceles* in North America, Central America, and the West Indies (Araneae, Loxoscelidae). Bulletin of the American Museum of Natural History175:264–360. http://digitallibrary.amnh.org/dspace/handle/2246/981

[B7] Gonçalves-de-Andrade RM Galati EAB Tambourgi DV (2001) Presença de *Loxosceles similis* Moenkhaus, 1898 (Araneae, Sicariidae) na Serra da Bodoquena, Estado de Mato Grosso do Sul, Brasil. Revista da Sociedade Brasileira de Medicina Tropical 34: 275–277. http://www.scielo.br/scielo.php?script=sci_abstract&pid=S0037-86822001000300008&lng=en&nrm=iso&tlng=ptdoi: 10.1590/S0037-86822001000300008 11460214

[B8] Gonçalves-de-Andrade RM Tambourgi DV (2003) First record on *Loxosceles laeta* (Nicolet, 1849) (Araneae, Sicariidae) in the West Zone of Sao Paulo City, São Paulo, Brazil and considerations regarding its geographic distribution. Revista da Sociedade Brasileira de Medicina Tropical 36:425–426. http://www.scielo.br/scielo.php?script=sci_arttext&pid=S0037-86822003000300019&lng=en&nrm=iso&tlng=en10.1590/s0037-86822003000300019 12908048

[B9] Gonçalves-de-Andrade RM Pretel FD Tambourgi DV (2007) The spider *Loxosceles adelaida* Gertsch, 1967 (Araneae, Sicariidae) in the Karstic area of Ribeira Valley, PETAR, São Paulo, Brazil. Journal of Entomology 4:46–50. http://scialert.net/qredirect.php?doi=je.2007.46.50&linkid=pdf

[B10] Martins R Knysak I Bertani R (2002) A new species of *Loxosceles* (Araneae:Sicariidae) of the *laeta* groupfrom Brazil (Araneae: Sicariidae). Zootoxa 94:1–6. http://www.mapress.com/zootaxa/2002/zt00094.pdf

[B11] Platnick NI (2011) The world spider catalog, version 11.5. American Museum of Natural History. Avaiable from http://research.amnh.org/entomology/spiders/catalog81–87/index.html

[B12] Ribera C Planas E (2009) A new species of *Loxosceles* (Araneae, Sicariidae) from Tunisia. ZooKeys 16: 217-225. doi: 10.3897/zookeys.16.232

[B13] Silveira AL (2009) Primeiro registro sinantrópico de *Loxosceles laeta* (Nicolet, 1849) (Araneae, Sicariidae) no Município do Rio de Janeiro, Estado do Rio de Janeiro. Revista da Sociedade Brasileira de Medicina Tropical 42:723–726. http://www.scielo.br/scielo.php?script=sci_arttext&pid=S0037-86822009000600021&lng=en&nrm=iso&tlng=ptdoi: 10.1590/S0037-86822009000600021 20209362

